# Criticality of the Self-Heating Effect in Polymers and Polymer Matrix Composites during Fatigue, and Their Application in Non-Destructive Testing

**DOI:** 10.3390/polym11010019

**Published:** 2018-12-23

**Authors:** Andrzej Katunin

**Affiliations:** Institute of Fundamentals of Machinery Design, Silesian University of Technology, Konarskiego 18A, 44-100 Gliwice, Poland; andrzej.katunin@polsl.pl; Tel.: +48-32-237-1069

**Keywords:** self-heating effect, composite structures, polymers, cyclic loading, vibrothermography, fatigue

## Abstract

The self-heating effect is a dangerous phenomenon that occurs in polymers and polymer matrix composites during their cyclic loading, and may significantly influence structural degradation and durability as a consequence. Therefore, an analysis of its criticality is highly demanding, due to the wide occurrence of this effect, both in laboratory fatigue tests, as well as in engineering practice. In order to overcome the problem of the accelerated degradation of polymer matrix structures, it is essential to evaluate the characteristic temperature values of self-heating, which are critical from the point of view of the fatigue life of these structures, i.e., the temperature at which damage initiates, and the safe temperature range in which these structures can be safely maintained. The experimental studies performed were focused on the determination of the critical self-heating temperature, using various approaches and measurement techniques. This paper present an overview of the research studies performed in the field of structural degradation, due to self-heating, and summarizes the studies performed on the evaluation of the criticality of the self-heating effect. Moreover, the non-destructive testing method, which uses the self-heating effect as a thermal excitation source, is discussed, and the non-destructivity of this method is confirmed by experimental results.

## 1. Introduction

The self-heating effect is one of the most dangerous phenomena during the operation of elements and parts of machines that are made of polymers and polymer matrix composites (PMCs) that are subjected to cyclic loading or extensive high-magnitude vibrations. This fact implies a necessity for the deep investigation of this effect, including its physical nature, the consequences, and the relations with degradation mechanisms in the above-mentioned types of structures, as well as the estimation of safe temperature ranges, which are important from the maintenance point of view for such structures, and the application of the self-heating effect for structural diagnostics purposes. Since the nature of the self-heating effect is connected with polymers, and is driven by the behavior of a polymer in PMCs, all further considerations relate to polymers and PMCs.

The physical nature of the self-heating effect relies on mechanical energy dissipation as a consequence of a phase lag *δ* between stress *σ*_0_ and strain *ε*_0_ magnitudes during cyclic loading. The stress and strain relations for a cyclically loaded structure can be presented as follows:

(1) $$\sigma =\sigma_{0}e^{i\omega t+i\delta }$$

(2) $$\varepsilon =\varepsilon_{0}e^{i\omega t}$$

where *ω* denotes the angular velocity, *t* denotes time, and *i* is an imaginary unit $\left(i^{2}=-1\right)$.

**Figure 1:**
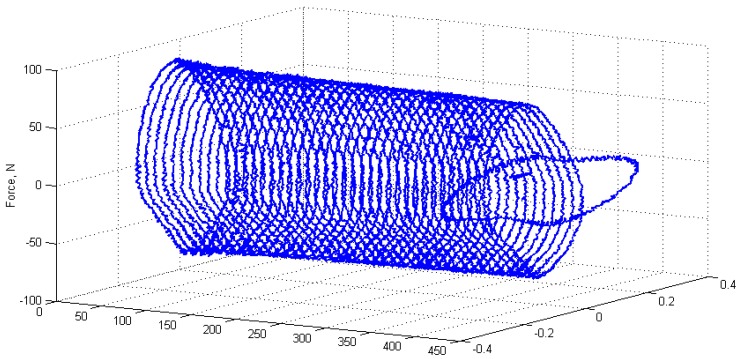
Experimental example of a hysteresis loop evolution during the appearance of the self-heating effect in a polymer matrix composite (PMC) structure under cyclic loading.

Such a phenomenon initiates an appearance of a mechanical hysteresis resulting from the viscoelastic nature of the most of industrial polymers and the composites based on them, which in consequence, result in energy excess. A schematic and experimental hysteresis loop for cyclically loaded polymers and PMCs can be found, e.g., in [1,2,3,4,5], while an exemplary hysteresis loop evolution during the appearance of the self-heating effect in a cyclically loaded PMC structure is presented in Figure 1 [6]. The change of geometry and the orientation of the last loop indicates structural failure.

**Figure 2:**
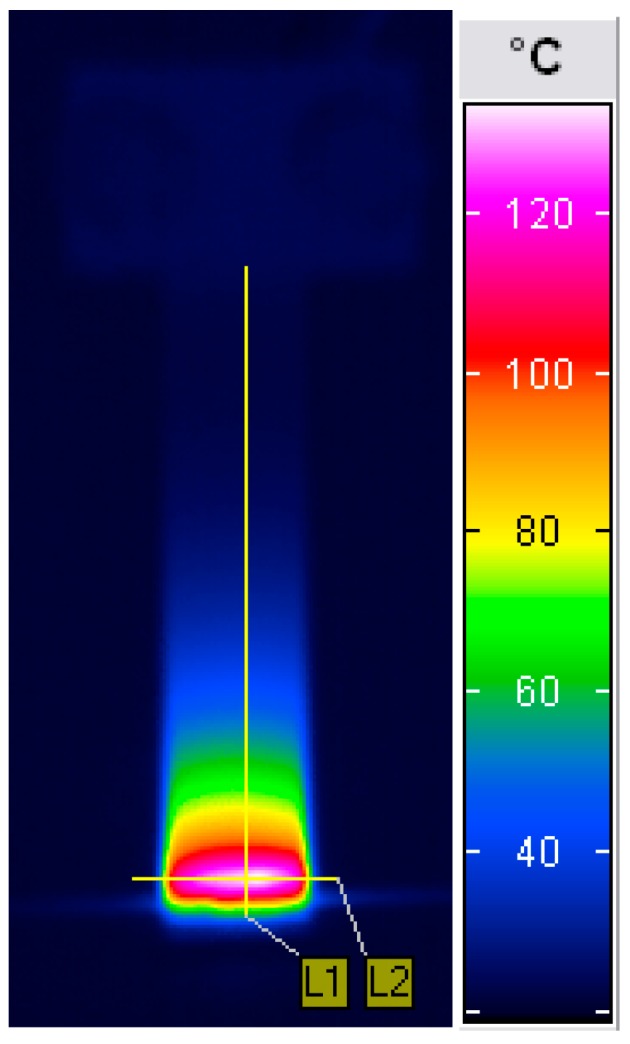
Typical self-heating temperature surface distribution in cantilever mode loading.

This mechanical energy is dissipated mostly in the form of thermal energy [1,7,8,9], while the rest of the energy is partly stored in the microstructure of the material [10], dissipated in the form of (micro-)plastic deformation [11,12], viscoplastic deformation, and isotropic and kinematic hardening [13]. The phenomenon of self-heating refers to the internal friction of the material on a molecular level [8], which leads to hysteretic behavior. Further, the heat resulting from the dissipative processes causes the increase of the temperature of the loaded structure, which is called the self-heating temperature. The temperature increase is particularly caused by the low thermal conductivity of most of the structural polymers and PMCs, especially thermoplastics [14], and may reach high values, exceeding the glass transition temperature of a polymer (see Figure 2), which was observed in numerous experimental studies, e.g., [6,15].

Using the approximate formulation of viscoelasticity [16], the phase lag *δ* can be presented in terms of complex material parameters; in particular, the complex modulus. Considering the influence of a self-heating temperature and its rate, the complex modulus can be represented by the following relation [17]:

(3) $$E^{*}\left(\omega ,T\right)=\frac{\sigma }{\varepsilon }=E^{\prime }\left(\omega ,T\right)+iE^{\prime \prime }\left(\omega ,T\right)$$

where:

(4) $$E^{\prime }\left(\omega ,T\right)=\omega \int_{0}^{\infty }E\left(t,T\right)\sin\omega t\;dt$$

(5) $$E^{\prime \prime }\left(\omega ,T\right)=\omega \int_{0}^{\infty }E\left(t,T\right)\cos\omega t\;dt$$

are storage and loss moduli, respectively, and *T* is temperature. The storage modulus describes the elastic behavior of a polymeric or PMC structure, while the loss modulus describes its viscous behavior. The connection of these moduli with a phase lag *δ*, and relations (1) and (2), is as follows:

(6) $$\tan\delta =\frac{E^{\prime \prime }\left(\omega ,T\right)}{E^{\prime }\left(\omega ,T\right)}.$$

**Figure 3:**
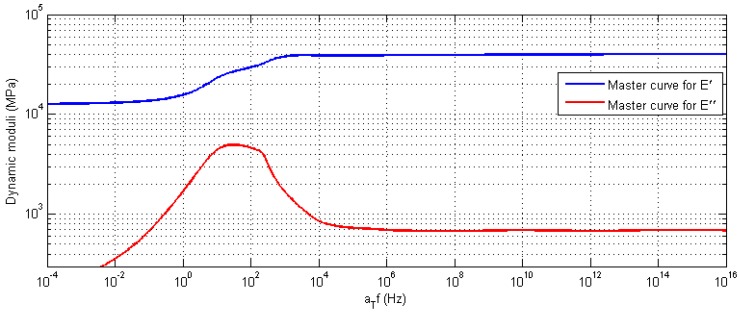
Exemplary master curves for the dynamic moduli for a glass fabric-reinforced PMC.

Assuming the linear thermoviscoelasticity in many industrial polymers that are used for construction purposes as well as matrices of PMCs, the behavior of such materials can be described by so-called master curves, which represent the dependency of a mechanical property (e.g., moduli) on a temperature and loading frequency. The construction of a master curve is based on the Arrhenius relation (or its modified version presented in [17]), and the time–temperature superposition principle. Detailed information on the measurements and further calculations necessary for the construction of master curves can be found in [17,18,19,20,21]. An example of such a master curve is presented in Figure 3.

The process of self-heating can be described by the following non-stationary equation of heat transfer [22]:

(7) $$c\left(T\right)\rho \left(T\right)\frac{\partial T\left(t\right)}{\partial t}-\nabla \left[\lambda \left(T\right)\nabla T\left(t\right)\right]=Q_{d}\left(t\right),$$

where *T(t)* denotes the temperature distribution over a polymeric or PMC structure at time *t*, *ρ(T)* is the density, and *c(T), λ(T)* are the thermal capacity and conductivity, respectively. The term *Q_d_(t)* in (7) can be considered as a source function, and represents the energy dissipation rate, i.e., the energy dissipated at time *t* defined as [22]:

(8) $$Q_{d}\left(t\right)=\frac{\omega }{2\pi }\int_{0}^{2\pi /\omega }\sigma \left(t\right)\frac{d\varepsilon \left(t\right)}{dt}dt.$$

The generation of energy *Q_d_(t)* is usually observed as the self-heating phenomenon, and its occurrence depends on various factors, including heat transfer between a loaded structure and ambient medium. Considering (7) and (8), as well as (3)–(5), the self-heating temperature evolution can be described by the following general relation (cf. [23]):

(9) $$\Delta \dot{T}=\frac{\omega E^{\prime \prime }\left(\omega ,T\right)\sigma_{\max}^{2}}{\pi \rho \left(T\right)c\left(T\right)}$$

which reflects a direct relation of the heat generation with the loss modulus $E^{\prime \prime }\left(\omega ,T\right)$.

The self-heating temperature distribution depends directly on the applied stress, and is usually non-uniform due to the non-uniform stress distribution during cyclic loading (see Figure 2). Similar temperature distributions can be found, e.g., in [24,25], while a typical distribution during cyclic loading is presented in [20,26,27,28] for tensile, in [29] for compressive, and in [20] for shear loading, respectively. The dependency between these two quantities is as follows: the higher the stress concentration, the higher the self-heating temperature. In contrast to metallic structures, the heat generation of a viscoelastic body is dominant [30,31], which may cause a significant increase in the self-heating temperature in a loaded structure.

**Figure 4:**
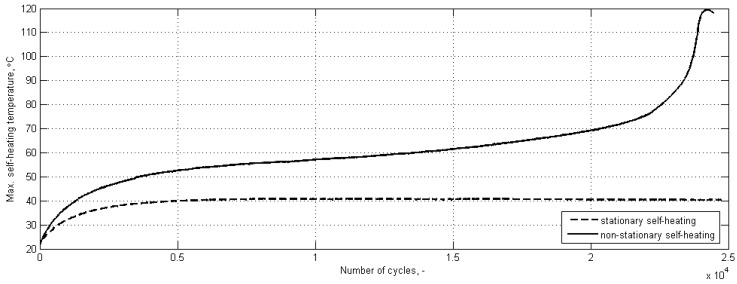
Physically possible scenarios of self-heating.

The self-heating effect may follow two physically possible scenarios: a stationary and a non-stationary one [32], which leads to mechanical and thermal fracture, respectively [33]. In the case of stationary self-heating, the self-heating temperature profile has two phases: the first phase of exponential character, governed by the second law of thermodynamics, and the second phase, when self-heating temperature stabilization occurs (see Figure 4).

This means that the self-heating temperature remains constant for a prolonged period. This stabilization is reached, due to the equilibrium between the generated thermal energy as a result of self-heating, and the thermal energy convected and radiated to the environment. A self-heating scenario depends on the level of applied stress. In the case of exceeding the critical level of the applied stress (which is usually on the level of 50–85% of the total fatigue life, depending on material and loading parameters [8,34,35,36]), the thermal equilibrium does not occur, and the second phase is characterized by a slope, but retains linear characteristic (see Figure 4). The self-heating effect in such a case follow the non-stationary scenario, and the appearance of a slope on the temperature history plot in the second phase is a result of mechanical degradation of a structure [6], which, in the end, results in the progressive development of damage in the form of voids and microcracks [37,38,39]. When these microcracks accumulate, the macrocrack appears, which in fact, ends the second phase of self-heating and initiates the third phase. This phase is characterized by a rapid character of development, since the process is dominated by the frictional heating in a macroscopic sense, i.e., the newly appeared surfaces in a propagating crack start to slide on each other under cyclic loading, which results in a rapid increase of a resulting temperature. In the case of the appearance of the third phase in non-stationary self-heating (called also the “thermal catastrophe” [40]), the resulting self-heating temperature values often reach or even exceed the glass-transition temperature of the polymers or polymeric matrices of PMCs [6,41,42,43]. The above-described three-phase model of non-stationary self-heating was mentioned in many other studies [8,26,38,44,45,46,47,48,49,50,51,52].

In this paper, the overview of research studies on the self-heating effect, and especially the studies focused on evaluation of the criticality of the self-heating effect based on the authored studies in the last few years were summarized. Moreover, based on the comparison of the determined values of the critical self-heating temperature, the safe temperature range for operation and non-destructive testing (NDT) were established. Finally, the newly developed NDT method for polymeric and PMC structures based on their self-heating was presented, together with the newest results on its damage detectability. This paper summarizes over 10 years of the author’s studies on the self-heating effect.

## 2. Literature Review on the Self-Heating Effect

The self-heating effect has been deeply studied in terms of the fatigue and degradation of polymeric and PMC structures, as well as their structural diagnostics for over 50 years. In this section, an overview on the most important research studies performed in this period is presented, and the mechanical and civil engineering problems where the self-heating effect appears, as well as its potential engineering applications, are discussed.

### 2.1. Self-Heating Effect—Historical Overview of the Research Studies

Although the viscoelastic and thermoviscoelastic behaviors of polymeric materials were extensively studied in the first part of the 20th century, the problem of self-heating started to be deeply investigated in the 1960s, due to its appearance in structural elements in solid rocket propulsion systems [53], and were focused mainly on the failure of polymeric structures [54,55]. These studies introduced a research interest to the self-heating effect of many scientific groups, which resulted in numerous publications in this thematic area in the 1960s. Due to its practical importance, numerous researchers were focused on the development of empirical fracture models of polymeric elements that were subjected to self-heating, in order to describe the phenomenology, and to predict this process. Such models were presented, in particular, in the studies of Tormey [54], as well as in numerous studies of Schapery (see e.g., [56,57]). 

In the same period, extensive studies on the self-heating effect in polymers and its influence on the fatigue and fracture of polymeric structures were started in USSR, firstly in Moscow [44,58], and then in the Academy of Sciences of Latvian [45,46] and Ukrainian [59] SSR. The first evaluations of an influence of the self-heating effect on polymer degradation and fracture had mostly phenomenological characters [44,45,46,58,60] and based on Zhurkov’s kinetic theory [61,62], the first attempts on analytical modelling of the self-heating effect in the light of the mechanics of continuous media were undertaken in the same period [59]. Independently, analytical modelling of the self-heating effect was introduced by Tauchert [63,64], where he proposed a coupled thermoviscoelastic model. In the 1970s and 1980s, extensive theoretical studies were established in the research group of Karnaukhov, Kirichok, and Senchenkov from the Institute of Mechanics of the Academy of Sciences of the Ukrainian SSR in Kiev, where the authors formulated exact solutions for many specific problems of self-heating, including critical state evaluation [40], and the exact solution for the self-heating appeared in viscoelastic solids of various geometrical properties, as well as anisotropy and material inhomogeneities [65,66,67,68,69,70,71]. These research studies were continued in the next years, which resulted in the formulation of the coupled theory of thermoviscoelasticity presented, e.g., in [72]. These studies are continued until nowadays, in which the mentioned theory was extended to thermoelectroviscoleasticity, introduced in [73], which considers a piezoelectric effect in polymers and PMCs (see e.g., [74,75,76,77]). In the latest period, numerous theoretical models of self-heating in polymeric and PMC structures were developed by other research groups. In particular, Molinari’s group developed quasi-static stationary self-heating models for cyclically compressed [78], twisted [79], and bent [80] polymeric structures. Increasing interest in the problem of modelling the self-heating effect in polymeric and composite structures is observed in the last decade, with a focus on the development of coupling between types of thermomechanical interactions. Several models of self-heating of viscoelastic structures were based on a thermodynamic framework (see e.g., [81,82]). Other recently developed models consider more complex behavior of polymers, including finite strain thermoviscoelasticity [27], thermoviscoelasticity within large deformations [20], thermoviscohyperelasticity [83], coupled viscoelastoplasticity [13,84,85,86], hygro-thermomechanical coupling [87], and others.

The developed theoretical models often constitute a basis for the development of fatigue models, considering the self-heating effect. Starting from simple phenomenological models proposed by Oldyrev and Tamuzh [45,46,61,88,89,90], the modelling of the fatigue and fracture of polymers and PMCs was successfully developed over the decades. As the authors of the previous studies have stated, the influence of the self-heating effect cannot be negligible during the fatigue of polymeric and PMC structures; therefore, extensive studies in the development of fatigue models were performed in past few decades and are developed until the present day. Numerous empirical fatigue models were developed based on experimental fatigue data; in particular, Bellenger et al. [91] fitted experimental *S*-*N* curves by a logarithmic relationship, which describes the progress of structural degradation; the authors of [92,93] developed a fatigue model for the prediction of a number of cycles to failure, taking into consideration stationary self-heating; a similar approach was used in [94], based on the Kachanov’s damage function, and considering stationary self-heating; while Naderi, Khonsari and Kahirdeh based their fatigue models on thermodynamic and acoustic entropy [48,95,96]. A comprehensive reviews on fatigue of short fiber reinforced PMCs were presented by Mortazavian and Fatemi [9,14]. Another approach of the evaluation of the fatigue degradation of polymeric and PMC structures is based on various types of damage accumulation functions [97,98], in particular, viscoelastic complex moduli. According to the studies of Miyano et al. [99,100] residual stress can be calculated, considering the thermoviscoelastic behavior of a PMC structure. Such an approach was used in [42], where the decrease of a normalized damage function was based on a temperature- and frequency-dependent storage modulus. Special attention is deserved for the newest studies of Shojaei et al. (see e.g., [4,101]), where the authors described the developed fatigue models for polymers, considering the self-heating effect in terms of the mechanics of the deformable medium, plastic interactions, and the anisotropy of the considered materials.

Besides the extensive theoretical investigation of the self-heating effect and its influence on the fatigue of polymeric and PMC structures, many important experimental works were performed, in order to investigate this effect. Important results were presented by Rittel in [7], where empirical proof on the thermomechanical coupling of the self-heating effect was given. Additionally, Rittel presented important conclusions on the conversion factors of mechanical to thermal energy during self-heating [7,41,102]. Later, the same research group investigated the mechanism of self-heating, and presented its molecular nature (chain mobility) in [8], which additionally confirmed the previously presented evidence on microstructural changes during self-heating [30,103]. A coupled thermomechanical response of a polymeric structure was analyzed and compared with a theoretical model, with special attention on defect formation in [38]. Bellenger et al. [91] performed experimental studies during the cyclic bending loading of PMCs, and presented the microstructure of the fatigue surface for thermal and mechanical fracture, showing different fracture mechanisms for these scenarios. Many important findings were reported by the team of Naderi, Khonsari and Kahidreh [26,51,95]. For the characterization of the structural degradation of polymeric and PMC structures subjected self-heating, they compared the thermal response of a tested structure with its acoustic emission (AE) response, in order to monitor degradation in the form of a number of acoustic events, or the amount of acoustic energy, as well as extensive microstructural characterization. At the same time, Dattoma and Giancane [104] performed tests by combining two measurement techniques—digital image correlation (DIC) and thermography, which allowed for the determination of heat sources representing the initiation of fatigue damage in tested structures. Recently, growing interest for the self-heating effect initiated in several specific studies, namely the characterization of self-heating and structural conditions during its occurrence at prestress conditions investigated by the group of de Lima [105,106], while the authors of [2] investigated the influence of humidity on self-heating in a polymer. Another valuable study was presented by Mares et al. [107], where the authors presented a model of self-heating of a viscoelastic structure with inclusions, and indicated that the temperature values that appeared at the interface may exceed decomposition temperature values.

In experimental studies in the field of the self-heating, the authors of this paper are focused on the evaluation of thermal failure of PMCs [6], their thermal responses during resonant vibrations [108,109], and recently, the multiphysical characterization of structural degradation of PMCs during the occurrence of the self-heating effect; in particular, the criticality of the self-heating effect, as well as the application of this effect to the non-destructive testing of polymeric and PMC structures, which is a subject of the next section of this paper.

### 2.2. Practical Problems Related to the Self-Heating Effect

The first practical problems that introduce intensive research studies on the self-heating effect were related to the structural degradation of polymeric elements of solid rocket propulsion systems. These viscoelastic elements were subjected to thermal stresses in the presence of non-uniform temperature fields [110]. One of the problems was also described by Tormey and Britton [54], where the viscoelastic solid propellant flowed out of a rocket engine because of cyclic vibration, resulting in softening of this propellant. This observation found an application in further studies of Loginov et al. [111,112], where the authors used the self-heating effect for the characterization of viscoelastic explosives. Further, systems for explosive detection based on the self-heating response of explosive materials and vapor pressure in sealants and bags were proposed by Miller, Woods and Rhoads [113,114]. The problem of self-heating also occurs in ultrasonic welding applications [115], where temperature needs to be controlled appropriately, or in composite high-speed driveshafts for automotive and rotorcraft applications [116].

From the beginning of the investigation of fatigue and vibrational testing of polymers and PMCs, the self-heating effect was one of the serious problems when fatigue life was under evaluation. Such a problem appears, e.g., in viscoelastic dampers that are widely used in mechanical and civil engineering. The generated heat influences the residual life of structural elements made of viscoelastic materials, due to the highly temperature-dependent moduli that represent the mechanical properties of such materials. The problem of self-heating in viscoelastic dampers has been reported in numerous studies (see e.g., [117,118]). Therefore, in order to avoid self-heating or at least to minimize its influence, various approaches were applied. In many cases, the loading frequency was significantly decreased (see e.g., [119]), which allows for the neglect of the self-heating effect, while another approach was based on cooling the surface of a tested structure subjected to fatigue loading [44,46,50,120,121]. The self-heating effect, however, can be utilized for the material characterization and diagnostic problems of polymeric and PMC structures.

Following the concept of the critical stress value and its relation to self-heating temperature introduced in [33], extensive studies on fatigue limit determination were introduced in the last decade, and actively developed in recent years [34,35,36,52,122,123,124,125,126,127]. In these studies, the critical stress value considered as an indicator of a self-heating effect stationarity, is referred to the fatigue limit value. In the mentioned studies, the self-heating effect is used for the rapid determination of the fatigue limit of polymers and PMCs. The self-heating effect has also found an application in the monitoring of adhesively bonded composite joints used in naval applications [29].

As was previously observed, the highest values of the self-heating temperature are noticeable in the region of the highest stress concentrations, which often indicate the locations of initiation of fracture. This property can be used for damage detection in polymeric and PMC structures, which were reported in numerous studies [1,24,128,129,130]. An overview on the self-heating based diagnosis of such structures is extended in further sections.

## 3. Criticality of the Self-Heating Effect

The concept of a criticality of the self-heating effect was introduced by Ratner, Korobov, and Agamalyan in [33], where it was defined as a critical stress at thermal fracture, and a critical temperature value appearing under this critical stress. The authors of [40] also mentioned in their paper the criticality of self-heating, and provided an analytical solution for its determination. Later, the criticality of the self-heating effect was specified by introducing a parameter that describes the character of an increase of the self-heating temperature; namely, the temperature value beyond which the self-heating temperature history profile becomes unstable [70]. Recent studies on the criticality of the self-heating effect are presented in [42], where the specific temperature value on the surface of a PMC specimen is subject to cyclic bending loading, namely the critical self-heating temperature value, is assumed as a measure of the degradation degree of a structure subjected to such a kind of loading. Later, Kahirdeh and Khonsari [131] proposed a similar criterion of criticality of structural degradation of PMCs, based on thermal and AE responses. In recent years, deep and extensive studies on the criticality of the self-heating effect were undertaken, in order to determine a safe temperature range, and thus, safe regimes of the operation of composite structures subjected to cyclic loading or vibrations.

All of the experimental studies presented below were performed on specimens manufactured from 14-layered epoxy-based PMC sheets reinforced by unidirectional E-glass fabric purchased from Izo-Erg S.A. (Gliwice, Poland) with a thickness of 2.5 mm, a width of 10 mm, and variable lengths from 70 to 100 mm, depending on the clamping conditions; however, the effective length, i.e., the length at which the loading was applied was similar in all studies. The mechanical properties, as well as the results of dynamic mechanical characterization of the tested PMC can be found in [17].

### 3.1. Determination of Critical Self-Heating Temperature

**Figure 5:**
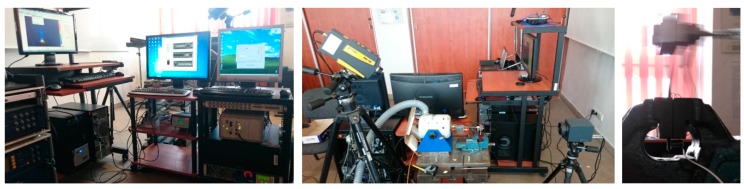
Laboratory test rig used for the determination of criticality of the self-heating effect.

The author’s earliest studies on the determination of a critical value of the self-heating temperature were based on an approximation of the history curves, using a double-exponential model. The mentioned history curves referring to the loading force history (related to a stress), vibration velocity history (related to a strain), and the self-heating temperature history appeared on the surface during cyclic bending of a PMC structure. The measurements were performed in an own-designed laboratory test rig, which loaded PMC specimens in cantilever bending mode. The pictures of the apparatus used for the performed tests are presented in Figure 5. The test rig was equipped with an electrodynamic shaker with amplifier for the excitation of a tested specimen, a force sensor, and an accelerometer used for monitoring and controlling the process, a laser Doppler vibrometer focused on a tested specimen for measuring vibration velocity, an infrared (IR) camera used for the registration of a thermal response of a tested specimen, and an AE system with a single sensor glued directly to a specimen in its non-loaded part (see Figure 5c). The details on the equipment used, as well as the measurement parameters, can be found in [132].

**Figure 6:**
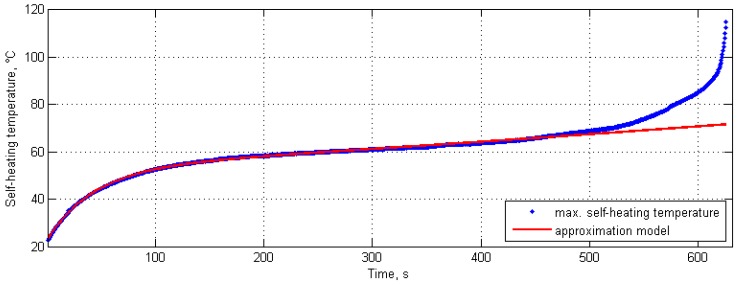
Exemplary self-heating temperature history curve with approximation model.

During the construction of the self-heating temperature history curves, the envelopes were considered for the force history and the vibration velocity history, and the maximal surface temperature was considered during the construction of the self-heating temperature history curve. The results of these studies can be found in [42,132,133]. The idea of the application of an approximation model is based on the observation of a divergence between a history curve and the double-exponential approximation model, i.e., such a divergence indicates the beginning of the third phase of self-heating, with intensive structural degradation and a rapid temperature rise in the non-stationary self-heating scenario (see Figure 4). An example of the application of the approximation model to a self-heating temperature history curve is presented in Figure 6.

According to the above-presented definition of the critical self-heating temperature, such a divergence was observed for the force and vibration velocity histories, and resulting self-heating temperature values at the moment of divergence between these history curves and approximation models were noted. The determined critical self-heating temperature values at the moment of divergence for these cases were 83.74 and 82.44 °C [132], respectively, which are quite high values with respect to the critical self-heating temperature values that are determined from the approximation of the self-heating temperature history curves directly (median: 61 °C) [134]. This points towards the low sensitivity of force and vibration velocity to the appearance of macro-scale fatigue damage that is observed at the beginning of the third phase of self-heating.

**Figure 7:**
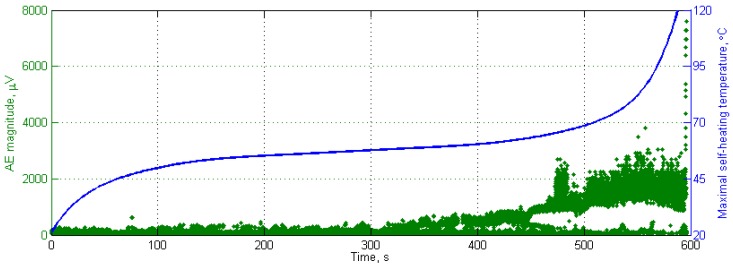
Representative acoustic emission (AE) response with the self-heating temperature history curve.

Further studies on the determination of the critical self-heating temperature were focused on AE measurements during the cyclic loading of PMC structures. These studies were based on the observation of AE events, which represent initial damaging processes, like matrix and fiber/matrix interface cracking, occurring during self-heating and the accompanying fatigue degradation of the tested structures. The performed studies covered the evaluation of various AE parameters and the selection of the parameters most sensitive to structural changes during self-heating [135]. The performed studies show that the most sensitive AE parameters to structural degradation are linear peak amplitude (ALIN), energy ratio (ETE), total energy of hit-cascade (CENY), threshold crossings (CCNT), and cascaded hits (CHIT). These parameters were named by the manufacturer of the Vallen^®^ measurement equipment used, and their detailed descriptions can be found in [132,135]. Further studies focused on the evaluation of criticality allowed for the determination of the critical self-heating temperature, respectively, for rapidly increasing the number of AE events (which indicate progressive cracking and the formation of a macrocrack) on the average level of 77–78 °C (with a minimal value of 67.2 °C) [132]. Finally, studies based on the clustering of AE events by the type of damage occurring allowed for the determination of respective critical self-heating temperatures for AE activity that are typical for macrocrack formation on the level of 65.73 °C [136]. The obtained results show that the results of the determination of criticality of the self-heating effect based on AE coincide with the results obtained from the approximation of self-heating temperature history curves (cf. Figure 6 and Figure 7).

**Figure 8:**
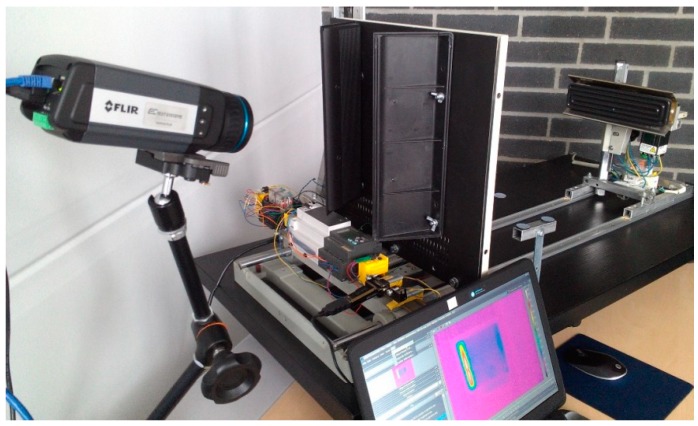
Laboratory test rig for measurements of thermal diffusivity [138].

Since the temperature measurements revealed good sensitivity to structural changes in a material, the evaluation of the self-heating effect criticality was supposed to be effective, based on the analysis of the thermodynamic processes appearing during self-heating. For this reason, numerous methods were used for the determination of the critical self-heating temperature. One of the approaches was based on the evaluation of differences of the thermal diffusivity of healthy and damaged regions of a PMC structure subjected self-heating [137]. The tested specimens were prepared on the laboratory test rig presented in Figure 5 in such a way that loading was applied until reaching a specific temperature value on its surface (since self-heating temperature distribution is non-uniform—see Figure 2 for instance—the maximal value of the self-heating temperature was considered). Due to the fact that specific temperature values can be related to a degree of structural degradation, by tabulating maximal self-heating temperature values, a set of specimens with various degree of degradation was obtained. The specimens prepared in such a way were then subjected to pulse heating using the test rig presented in Figure 8, which was used for the uniform heating of a specific region of a specimen by an IR radiator (see [137] for details). The thermal response was registered by an IR camera.

**Figure 9:**
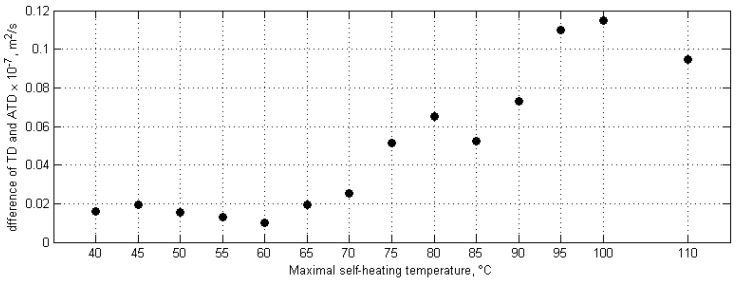
Relative apparent thermal diffusivity (ATD) for various self-heating temperature values.

The resulting thermograms were used for the determination of thermal diffusivity following the method presented by Parker et al. [139]. The relative apparent thermal diffusivity (ATD) was determined as follows: thermal diffusivity (TD) was evaluated for a healthy region, and a damaged region (called here the apparent thermal diffusivity, since the thermal diffusivity was determined for a PMC structure and air in gaps resulting from fracture). The relative ATD defined in [137] was then calculated as the difference between the TD and ATD. The obtained results for the difference between healthy and damaged regions for particular specimens with various degree of degradation due to self-heating are presented in Figure 9.

The results presented in Figure 9 clearly show that below the maximal self-heating temperature of 70 °C, the values of the relative ATD are comparable, while at 75 °C, a sharp increase of its value can be observed. Considering these results, it can be concluded that by using the method based on the evaluation of relative ATD, the critical self-heating values are in the range of 70–75 °C.

**Figure 10:**
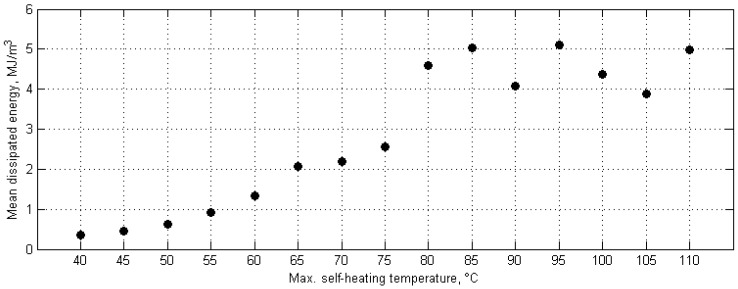
Amount of dissipated energy for various self-heating temperature values.

Another developed approach is the evaluation of the criticality of the self-heating effect based on thermodynamic response of a structure, used heat dissipation rate as a measure of structural degradation [140]. The experimental data were collected during the same fatigue tests as for the study of determination of apparent ATD; however, in this case, after reaching a specific maximal self-heating temperature of a tested structure, and removing cyclic loading, an IR camera registered the convectional cooling process of a structure. The idea of this approach is based on the thermodynamic equivalence between generated and dissipated thermal energy, which makes possible the determination of the amount of dissipated mechanical energy during self-heating, which can be an indicator of structural degradation. The method was firstly proposed for the evaluation of the residual life of steel [141] and then PMC structures [34] by Meneghetti and Quaresimin. This approach was used for the evaluation of fatigue damage of PMC structures [52,104,142], and then adapted by Naderi et al. [51] for the characterization of progressive damage in PMC structures subjected to fatigue loading accompanied with self-heating. According to this approach, collected self-heating temperature history curves after loading removal were approximated by using a linear regression model, which makes possible the determination of the amount of dissipated energy from the slope of a regression line. The performed calculations allowed for the determination of the heat dissipation rate for particular values of the self-heating temperature, which correspond to various degrees of structural degradation. The results of this study are presented in Figure 10.

As one can observe, the obtained results indicate a sudden change of amount of dissipated energy after exceeding 75 °C, which can be considered here as a critical value of the self-heating temperature.

**Figure 11:**
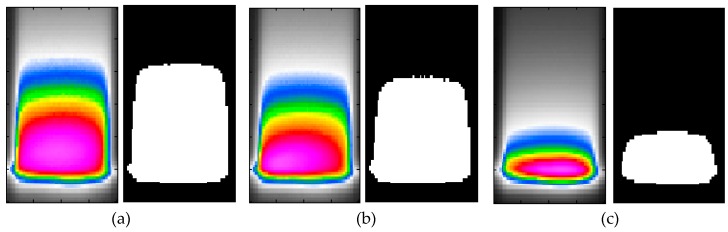
Thermograms of a specimen subjected self-heating, and their corresponding binarized versions: (**a**) before starting structural degradation, (**b**) after the initiation of structural degradation, (**c**) at structural failure.

The next approach of determination of the critical self-heating effect was based on the analysis of the variability of temperature distribution during cyclic loading, which changes the heated area due to the formation of a macrocrack at the beginning of the third phase of self-heating [143]. The changes of a heated area are connected with the structural degradation and the redistribution of stress—during the formation of a macrocrack, stress concentrates on this crack, while at more distant regions from a clamp, the stress become lower, accordingly. Such a phenomenon can be observed from a comparison of the distribution of temperature at different stages of degradation (see Figure 11). In this figure, three pairs of images representing the degradation evolution are presented: in each pair, the first image is a thermogram, while the second one is its binarized version obtained by a squeezing operation on the red color channel, and then subjected adaptive thresholding.

**Figure 12:**
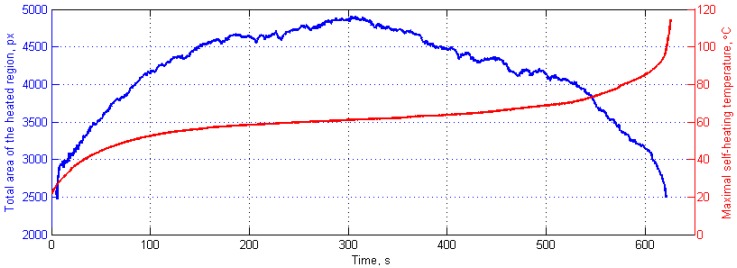
The evolution of a total area of a heated region with the corresponding maximal self-heating temperature history.

Such a change of heated area can be used as an additional measure of the criticality of the self-heating effect, since stress redistribution during progressive degradation also redistributes a surface temperature, due to the relation between these parameters (see Section 1 for more details). The analysis was performed on sequences of thermograms collected during fatigue tests, which were processed using the above-described procedures, and for the resulting binary images, an area of white region was calculated. Exemplary results of variability of heated areas, together with the corresponding maximal self-heating temperature for a selected specimen, are presented in Figure 12.

The curve of evolution of a total area of a heated region presented in Figure 12 shows characteristic increase, which can be explained by the heat diffusion processes in a healthy structure. At the 303rd second in Figure 12, one can observe that this curve reaches a peak, and then start monotonically decreasing. This decrease can be explained by the starting mechanical degradation and the formation of a crack, which causes the redistribution of a stress. In this study, the temperature corresponding with a peak value of a total area of the heated region was considered as a critical self-heating temperature. The performed statistical studies, based on the results from 36 specimens [143] allowed for the determination of a median value of the critical self-heating temperature obtained with this approach, equaling 61 °C.

**Figure 13:**
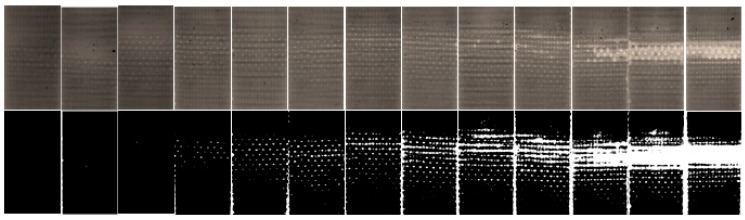
Exemplary set of microphotographs and the results of pre-processing for specimens subjected to self-heating, starting from 40 to 100 °C, with a step of 5 °C.

A set of specimens prepared in the same way as for the thermal diffusivity tests described above was subjected to microscopic testing in order to analyze surface cracks at various stages of degradation. The results of these studies were described in [39,132]. The criterion for the determination of the critical self-heating temperature value in this approach was based on the analysis of evolution of a crack density parameter, calculated as the number of cracks per unit of area [144] for specimens with various degrees of degradation. Before the determination of crack density, the obtained microphotographs were pre-processed by using unsharp masking (for sharpening) and global image thresholding (for binarization). The obtained binary images were normalized in order to obtain dimensionless crack density measures. The selected results of the original and pre-processed microphotographs are presented in Figure 13, which show the evolution of a degradation of PMC specimens subjected to self-heating. 

**Figure 14:**
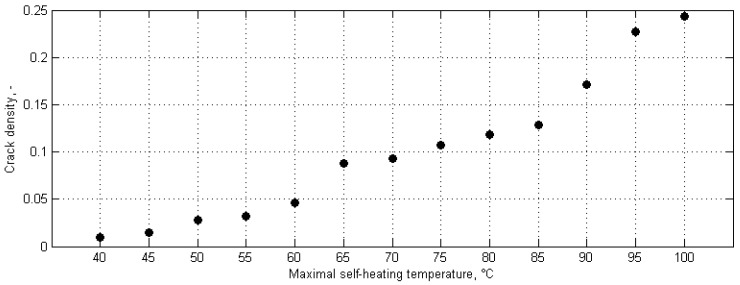
Crack density for various self-heating temperature values.

From the visual analysis, one can observe the formation of a through-the-width crack (which can be considered as a macrocrack) at ca. 70–75 °C. In order to obtain quantitative results, the determined crack density parameter was plotted vs. the maximal self-heating temperature, and a sudden change of a value of the crack density parameter was assumed as a criterion of the criticality of self-heating (see Figure 14). In this plot, one can observe the non-monotonic increase of a crack density parameter at 65 °C, which indicates abnormal structural changes, i.e., starting of progressive cracking. According to these results, the mentioned corresponding temperature was assumed as a critical self-heating temperature in this approach.

**Figure 15:**
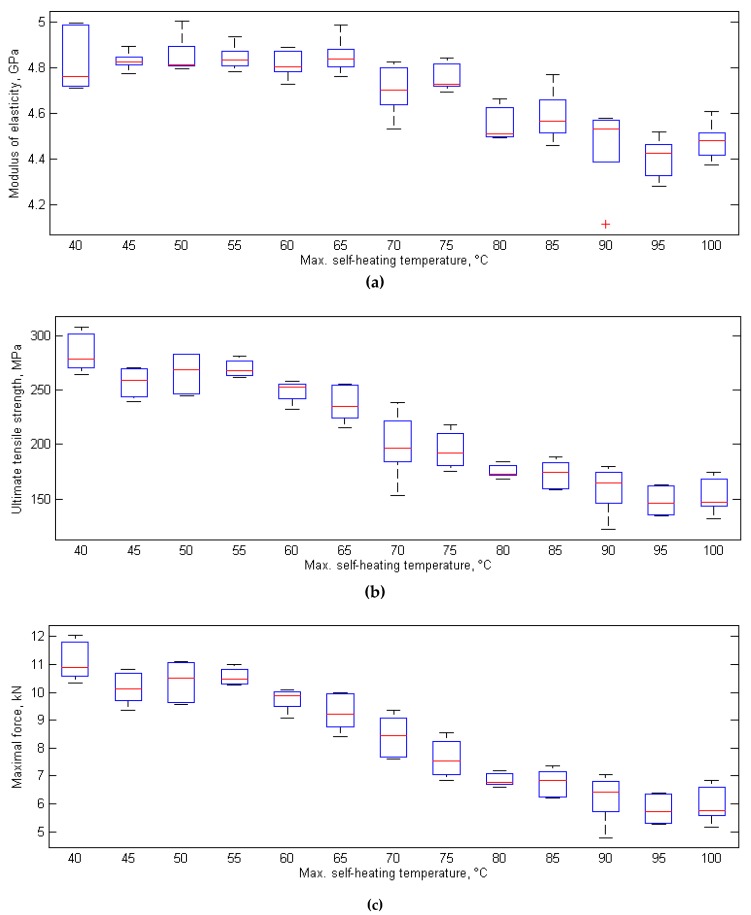
Results of destructive tensile tests for various self-heating temperature values.

The criticality of self-heating was also determined in destructive tensile quasi-static tests, which were performed on a set of specimens with variable degradation degrees prepared in the same way as for the thermal diffusivity tests [132]. Three parameters were determined in this study: residual elastic modulus, ultimate tensile strength, and maximal force at failure. The results of these tests presented vs. the maximal self-heating temperature are shown in Figure 15 in the form of bar plots, where the central marks denote median values obtained from five tests of specimens with same degradation degree, while the upper and lower edges of the boxes are the 25th and 75th percentiles, and the whiskers extend to the most extreme data points.

The obtained results for the residual elastic modulus (Figure 15a) indicate a sudden drop at 70 °C of the maximal self-heating temperature, while the results for ultimate tensile strength and maximal force at failure indicate such a drop at 65 °C. These drops allow the conclusion of the high advancement of structural degradation at the mentioned self-heating temperature values, since the fracture mechanism has developed so far that it influences the strength properties of the tested PMC specimens, and thus, these drops can be considered as a measure of degradation criticality. Following this, the mentioned corresponding maximal self-heating temperature values can be considered as the critical self-heating temperature values for this approach.

**Figure 16:**
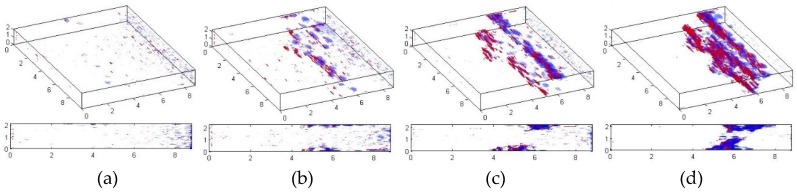
Selected processed tomograms for various self-heating temperature values: (**a**) 60 °C, (**b**) 75 °C, (**c**) 90 °C, (**d**) 105 °C.

In order to investigate the fracture mechanisms occurring during the fatigue of PMCs accompanied with deep self-heating, and to analyze their influence on the criticality of a degradation process, an additional study was performed on a set of specimens with various degrees of degradation, prepared in the same way as the previous usage of X-ray Industrial Computed Tomography (ICT) [145]. The X-ray ICT tests allowed for 3D representations of fractured specimens to be obtained, and observation of the initiation of cracking due to self-heating. The collected tomograms were subjected to pre-processing, in order to prepare them for further analysis by using the developed image processing procedure, which covered the application of a Gaussian filter and adaptive thresholding, based on the mean of the local intensity distribution. Next, the texture-based segmentation algorithms were applied in order to identify and classify cracks and delaminations. The selected results of the processed tomograms are presented in Figure 16. The full set of processed tomograms can be found in [145].

**Figure 17:**
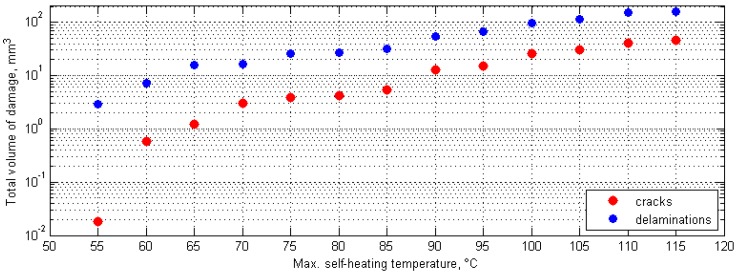
Total volume of damage for various self-heating temperature values.

To analyze the obtained results from X-ray ICT tests quantitatively, the obtained 3D images were rescaled to the specimens’ dimensions, and the total volume values for cracks and delaminations were calculated for each considered value of the maximal self-heating temperature. The evaluation was commenced from the value of 40 °C of the maximal self-heating temperature; however, the first structural changes were observed at 55 °C; therefore, the self-heating temperature values on the plots representing mentioned dependencies started from 55 °C (see Figure 17). The values in Figure 17 presented median values from four tests of specimens with the same degradation degree.

Analyzing the results of the X-ray ICT study, one can observe a rapid increase in the total volume of cracks at 60 °C of the maximal self-heating temperature, which was caused by the initiation of structural degradation. Below this value of the self-heating temperature, the total volume of cracks was on the level of scanning and processing errors, while delaminations were undetectable. This allows for an assumption that the value of 60 °C could be considered as the critical self-heating temperature in this study. This value was also confirmed in morphological analyses performed by using a scanning electron microscope (SEM) on the same specimens [146].

**Figure 18:**
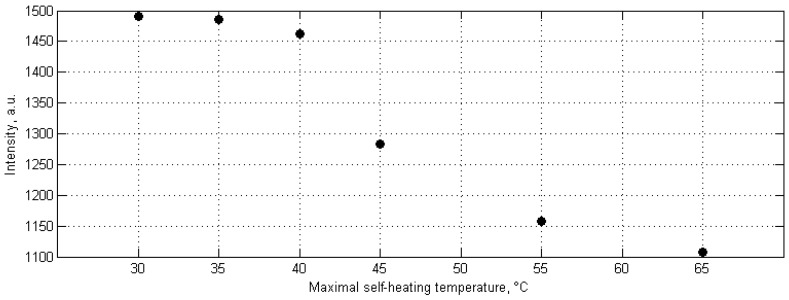
Intensity of Raman epoxy band (1256 cm^−1^) for various self-heating temperature values.

Finally, the analysis of the chemical degradation of tested PMCs during fatigue accompanied with self-heating was performed. The initial studies on chemical degradation were focused on the evaluation of residual cross-linking of a polymer used for a matrix of a tested PMC [147], initiated after observing the differences in evolution curves during the repeated cyclic loading of the same structure subjected to non-stationary self-heating [43]. The analysis of chemical degradation in the mentioned study was based on the evaluation of Raman spectra in the location of the highest mechanical stress concentration for a set of specimens with variable degradation degrees, prepared in the same way as that described previously in the temperature range of 30–65 °C. As a measure of chemical degradation, the Raman band of 1256 cm^−1^ was selected, since it is characteristic for epoxy ring backbone vibrations [148]. The obtained results of this study are presented in Figure 18. One can observe a sudden drop at 45 °C of the maximal self-heating temperature, which is related to a residual cross-linking of epoxy being a matrix of a tested PMC. Further studies on the chemical degradation of PMCs subjected to fatigue accompanied with self-heating [146] show that, in general, self-heating has negligible influence on chemical degradation in the temperature range characteristic for the appearance of structural degradation determined using previous approaches. The mentioned residual cross-linking does not influence structural integrity at lower temperatures. The chemical changes resulting from self-heating that affect the mechanical properties of PMCs, e.g., causing the permanent complex destruction of their matrix, were observed at self-heating temperature values of higher than 80 °C, which were confirmed both by Raman and FTIR spectroscopy studies.

### 3.2. Evaluation of the Safe Self-Heating Temperature Range

**Figure 19:**
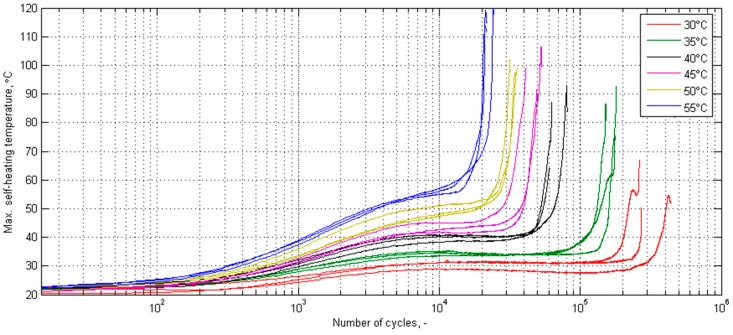
Selected self-heating temperature history curves for various temperature stabilization values.

Extensive studies on the evaluation of the criticality of the self-heating effect described in the previous subsection need to be summarized, in order to evaluate the most sensitive methods for the determination of this characteristic temperature, as well as to define safe self-heating temperature ranges, which allow for the operation and non-destructive testing of polymeric and PMC structures. The newest studies focused on the evaluation of the influence of the self-heating temperature on the residual life of such structures indicated that even a small increase of a self-heating temperature may influence the residual life of a structure significantly [149]. The performed experiment assumed the cyclic loading of PMC specimens, with various loading force values adjusted in such a way that the self-heating temperature on the surface stabilized onto a certain value. The results of this study are presented in Figure 19. One can observe that a temperature values increase from 30 °C to 33 °C may shorten the residual life of a PMC structure by two-fold or more, which was confirmed both by the analysis of a thermal response, as well as an AE response [32,149].

Nevertheless, the short-time exposition of polymeric and PMC structures to self-heating, when the loading has no fatigue character, has no consequences in structural degradation when the applied stress, and thus, the resulting self-heating temperature is not high enough to initiate the degradation processes, i.e., it does not exceed the critical self-heating temperature value. Numerous approaches used for the determination of these critical values presented in Section 3.1 need to be summarized and compared. For this purpose, the determined critical values in these studies are stored in Table 1.

From the results stored in Table 1, one can conclude that the initiation of structural degradation, considered here as the formation of damage that affects the mechanical properties of a structure subjected to self-heating, take place at the maximal self-heating temperature value appearing on a surface of 60 °C. Some studies described above give ambiguous results, such as detected cracks and delaminations on the level of measurement and processing error for a self-heating temperature of 55 °C in X-ray ICT studies, or the decreasing intensity of a Raman band of an epoxy group for a self-heating temperature of 45 °C, which has no influence on the mechanical properties of a loaded structure, which was confirmed in further tests. Considering also the specificity of degradation mechanisms observed during progressive cracking under the influence of self-heating, for the evaluation of a safe temperature range, two factors need to be taken into account: the location of damage initiation and a volumetric heat distribution. As reported by Oldyrev [46], the self-heating temperature is slightly higher inside a structure subjected to self-heating than on its surface, which was confirmed during recently performed microscopic studies [146]. This difference can be explained by the low thermal conductivity of industrial polymers in general, which causes heat to be stored inside the structure. The tomographic tests of specimens performed at various stages of degradation due to self-heating [145] confirmed that the initiation and propagation of damage starts on the upper and lower surfaces (see Figure 16), which is a result of the highest tension–compression stresses on the surfaces of a cyclically bent structure. Considering a fully reversed bending mode, the stresses in the midplane of a cyclically loaded structure are the lowest in the *z*-coordinate (thickness direction). This means that locations of a crack initiation and the maximal volumetric self-heating temperature values do not coincide with each other, and the correction of the self-heating temperature determined on the surface should not consider these phenomena as a coupled problem. From the experimental studies presented in [46], for a PMC structure, one can conclude that the difference between the surface and internal temperature values (in the center of a cross-section) remain constant when self-heating is in a stationary regime. According to the presented results in the above-cited study, this difference can reach from several to almost 15 °C, depending on the thickness of a structure, its thermal conductivity, and its loading parameters. In the case of the PMC structures tested using the above-described approaches, this difference may reach up to 10 °C, due the small thickness. Considering the results of a determination of the criticality of self-heating temperature, as well as the above discussion, the upper limit of the maximal self-heating temperature observed on the surface of a structure can be assumed to be 45 °C. This means that self-heating up to this temperature value for short periods does not cause the mechanical degradation of a loaded structure.

## 4. Self-Heating-Based Vibrothermography

The determined safe range of the self-heating temperature is an important parameter for the developed NDT method, which uses the phenomenon of self-heating for thermal excitation of the inspected polymeric and PMC structures. The self-heating effect was used for NDT purposes in numerous applications, e.g., for NDT damage characterization in carbon fiber-reinforced polymers (CFRP) [1,24]. The self-heating effect was also mentioned in the context of NDT of polymeric and PMC structures in [31,150,151], in terms of classical vibrothermography.

**Figure 20:**
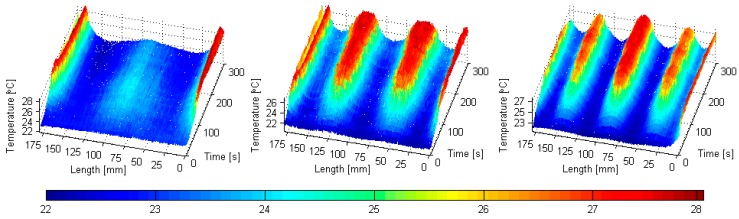
Evolution of longitudinal self-heating temperature profiles of PMC beams subjected to vibration at three first-resonant frequencies.

The observations of the characteristic temperature distributions during the cyclic loading of PMC structures at resonant frequencies [108,109] induced the author of this paper to develop a NDT method, which will use low-frequency resonant vibrations for the local thermal excitation of structures that make possible damage detection and identification. Based on these results, the new NDT method was developed, which was called self-heating-based vibrothermography (SHVT), due to its connection with the self-heating effect [152]. The proposed method consists of two main steps: in the first step, a modal analysis of a tested structure is performed to collect information about natural frequencies of this structure with their corresponding mode shapes; in the second step, the selected harmonics representing resonances (useful from the point of view of location of regions of maximal deflections during vibrations on resonant frequencies) are combined in a multi-harmonic signal, which is further used for the mechanical excitation of a structure in order to excite self-heating; the resulting thermal response is registered by an IR camera. Excitation with a multi-harmonic signal has a crucial importance in this method, since, as it was reported in previous studies (see e.g., [109,152]), the thermal response is directly connected with an excited mode shape (see examples in Figure 20) due to the relation between stress and self-heating temperature, as described previously in Section 2. This means that excitation with a single resonant frequency results in local heating in a location corresponding to the highest magnitude of vibration at this resonant frequency. In order to excite the whole surface of a tested structure, a simultaneous excitation at multiple resonant frequencies is necessary, which is possible by combining resonant harmonics into a composite excitation signal.

**Figure 21:**
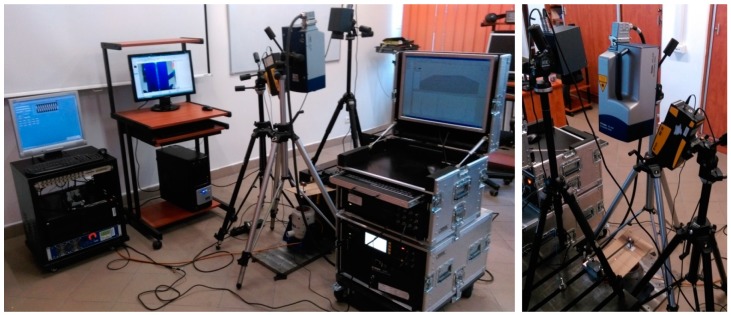
Testing apparatus for performing self-heating-based vibrothermography (SHVT) tests.

**Figure 22:**
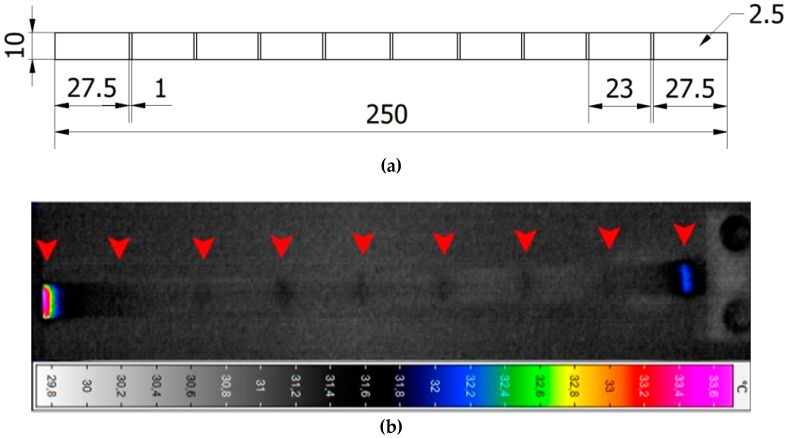
Exemplary results of damage identification by using the SHVT method: (**a**) scheme of the introduced damage; (**b**) thermal response of a PMC structure.

The analysis of the thermal response of a tested structure excited with a multi-harmonic signal allows for the detection and localization of disturbances in a temperature distribution, and its evolution that corresponds with flaws and damage locations in a structure. The testing apparatus used for SHVT is presented in Figure 21. It consists of two laser Doppler vibrometers and an electrodynamic shaker with an amplifier for performing modal analysis, and an IR camera for registering a thermal response during the second step of analysis. The excitation was performed by using the same electrodynamic shaker, and the control of the whole experiment was performed from three PCs. The detailed information about measurement equipment can be found in [152,153]. The exemplary results of damage identification in PMC elements are presented in Figure 22, together with a scheme of introduced damage. One can observe that all of the introduced damage sites were identified properly; however, their visibility is low, which may cause difficulties in their evaluation. For this purpose, the methods of the enhancement of thermograms were developed and adapted to increase the detectability of damage.

**Figure 23:**
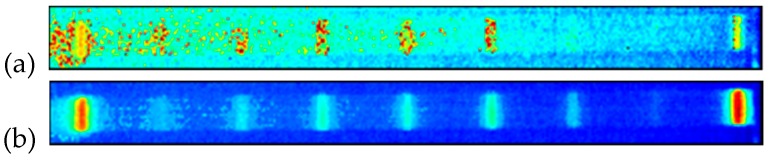

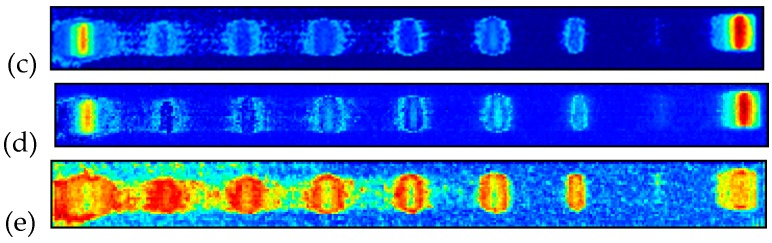
A single raw thermogram (**a**) and the exemplary results of the enhancement of damage detectability in the PMC elements of thermograms collected during SHVT inspection using: median value calculation (**b**), standard deviation value calculation (**c**), principal component thermography (**d**), undecimated discrete wavelet transform (**e**).

The exemplary results of the enhancement of damage identification ability are presented in Figure 23. The results of the enhancement are presented for the case shown in Figure 22; the enhancement methods cover simple statistics-based methods, as well as advanced methods, like thermographic signal reconstruction, partial least squares regression, and principal component thermography, Fourier and wavelet transforms, and others, are dedicated and widely applied for thermograms (see e.g., [154,155,156,157]). The results presented in Figure 23 clearly show the enhancement of damage detectability, with respect to the raw thermogram presented in Figure 22, and its transformed version presented in Figure 23a.

In order to evaluate the effectiveness of this method, the sensitivity to damage was analyzed on specimens with flat-bottom holes of various depths [153]. It was found that the damage (located in the opposite side to a tested surface) is detectable, starting from 0.75 mm of depth for the thickness of a tested element of 2.5 mm. For enhancing the detectability of damage, numerous image processing methods were developed and applied to the resulting sequences of thermograms, which resulted in the increase of the sensitivity of SHVT to various types of damage occurring in polymeric and PMC structures.

## 5. Conclusions

This paper presents phenomenological fundamentals on the appearance of the self-heating effect in viscoelastic polymeric and PMC structures subjected to cyclic loading, and an overview of the most important research studies on the self-heating effect, both theoretical and experimental, for a period of over 50 years. An important discussion in this paper is connected with the analysis of occurrence of the self-heating effect in engineering practice, as well as the application of this phenomenon in various research studies, with an emphasis on NDT and damage detection and characterization.

The paper also presents the newest research results on the determination of the criticality of the self-heating effect, using numerous methods based on the analysis of thermal and mechanical responses, acoustic emission, and microscopic, spectroscopic, and tomographic characterization. Such a versatile approach allows for the evaluation of the sensitivity of the applied methods to damage initiation, caused by the self-heating effect, and most importantly, obtaining verified and valid values of the critical self-heating temperature, considered here as a temperature of initiation of a mechanical damage that affects the mechanical properties of a structure. The results of the performed studies show that the lowest critical self-heating temperature was ca. 60 °C; however, considering the non-uniform temperature distribution inside loaded structures, it was assumed that this temperature can be lowered to 45 °C. The performed tests made it possible to determine the safe self-heating temperature range, and confirm the non-destructivity of the developed SHVT method.

Finally, a concept and the performance of the SHVT method was presented and discussed, with emphasis on damage detectability in the PMC structures, and its enhancement, using dedicated image processing methods. Further work is currently in progress, and focuses on the development of a tool for the effective enhancement of damage detectability in thermograms obtained by using the SHVT method.

## Figures and Tables

**Table 1:** Comparison of the critical self-heating temperature values determined using various approaches.

No.	Approach	Determined Critical Self-Heating Temp., °C
1.	Approximation of loading force history curves	83.74
2.	Approximation of vibration velocity history curves	82.44
3.	Approximation of self-heating temperature history curves	61
4.	AE activity	77–78
5.	Clustering of AE events	65.73
6.	Relative ATD	70–75
7.	Heat dissipation rate	75
8.	Variability of self-heating temperature distributions	61
9.	Surface crack density	65
10.	Residual elastic modulus in quasi-static tensile tests	70
11.	Ultimate tensile strength in quasi-static tensile tests	65
12.	Maximal force at failure in quasi-static tensile tests	65
13.	X-ray ICT	60
14.	Morphological analysis (SEM)	60
15.	Residual cross-linking using Raman & FTIR spectroscopy	80
